# SimArray: a user-friendly and user-configurable microarray design tool

**DOI:** 10.1186/1471-2105-7-102

**Published:** 2006-03-01

**Authors:** Richard P Auburn, Roslin R Russell, Bettina Fischer, Lisa A Meadows, Santiago Sevillano Matilla, Steven Russell

**Affiliations:** 1 Department of Genetics, University of Cambridge, Cambridge, UK

## Abstract

**Background:**

Microarrays were first developed to assess gene expression but are now also used to map protein-binding sites and to assess allelic variation between individuals. Regardless of the intended application, efficient production and appropriate array design are key determinants of experimental success. Inefficient production can make larger-scale studies prohibitively expensive, whereas poor array design makes normalisation and data analysis problematic.

**Results:**

We have developed a user-friendly tool, SimArray, which generates a randomised spot layout, computes a maximum meta-grid area, and estimates the print time, in response to user-specified design decisions. Selected parameters include: the number of probes to be printed; the microtitre plate format; the printing pin configuration, and the achievable spot density. SimArray is compatible with all current robotic spotters that employ 96-, 384- or 1536-well microtitre plates, and can be configured to reflect most production environments. Print time and maximum meta-grid area estimates facilitate evaluation of each array design for its suitability. Randomisation of the spot layout facilitates correction of systematic biases by normalisation.

**Conclusion:**

SimArray is intended to help both established researchers and those new to the microarray field to develop microarray designs with randomised spot layouts that are compatible with their specific production environment. SimArray is an open-source program and is available from .

## Background

The full utility of the spotted microarray format is clearly reflected in the range of its applications. Transcriptome arrays, containing cDNA, gDNA, or oligonucleotide probes, are used to measure differential gene expression [1-5]. Whole-genome arrays, typically composed of tiled gDNA or oligonucleotides [6], have been used to identify *in vivo *sites of protein-DNA interactions [7,8] or allelic variation [9,10]. Whilst these applications dominate, other formats, for example antibody arrays, facilitate analysis of protein and small-molecule analytes [11,12]. Thus, spotted microarrays enable high-throughput, cost-effective, and large-scale analysis of molecular interactions.

Robotic spotters deposit probes as an ordered array by repetition of a simple multi-step procedure [13-15]. First, the print tool is positioned over the first batch of probes to be printed, arranged in 96-, 384- or 1536-well microtitre plates. Second, the spotting pins are filled by capillary action with probe material. This step is often called a source visit. Third, the probe material is deposited on chemically-modified glass slides [14,16]. Finally, the pins are cleaned, to prevent cross-contamination between subsequent spot depositions, before re-filling and printing the next batch of probes. The diversity of instrumentation, spotting pins, and reagents available, mean that procedures may be refined for optimal throughput, spot density, morphology, and consistency [16]. Whilst this facilitates production of high-quality arrays, it can also lead to significant differences between facilities with regard to the instrumentation, protocols, and reagents employed.

Robotic spotters are supplied with sophisticated software to convert operator inputs to the precise list of instructions needed by the arrayer, *e.g*., how often each source visit is to be printed, and at which spot location [13-15]. Most spotters, however, are not supplied with adequate array design tools. Operators are instead left to develop suitable spot layouts in an *ad hoc *fashion. This oftenleads to sub-optimal designs with spots positioned according to print order, thus juxtaposing replicates, when a non-sequential or randomised spot layout can help to control for confounding spatial effects [17,18]. For example, biases caused by inconsistent probe concentrations in the microtitre plates [18,19] and local variations in hybridisation or washing efficiency [20-23], also see Additional file 1. Whilst random noise can be overcome with simple replication and averaging, systematic biases must be specifically addressed by randomisation and normalisation [24-27].

There is therefore a need for a microarray design tool that can generate randomised source visits and, in the case of some instruments, permit the use of variable numbers of replicates, since current spot density constraints and the need for genome-wide coverage mean that replication is often limited to the normalisation controls. Exogenous or 'spike' controls, *i.e*., probes that are complementary to targets not present in the genome of interest, can be employed for this purpose [28,29]. Print time and maximum meta-grid area estimates enable users to evaluate the suitability of the array design.

We have addressed the current lack of such microarray design tools by developing SimArray, a user-friendly and user-configurable program that generates a randomised spot layout, computes the maximum meta-grid area, and estimates printing time, in response to user-defined design decisions. The user enters these parameters by running SimArray twice. The first run produces the source visit list that can be edited to include variable numbers of replicates, or for specific source visits to be omitted when plates are partially filled. The second run processes this source list to create the spot layout, maximum meta-grid area and estimated print time. User-configurable files mean that SimArray can be adapted to most production environments.

## Implementation

SimArray was developed in Perl version 5.6.1 and 5.8.3, under both Windows and UNIX operating systems. SimArray can be run under Windows (after installing Perl; for example, ActivePerl [30]) and UNIX.

### Before the first run

Download the three configuration files and an 'index.sa' file from the SimArray web site [31]. Configuration files that describe instrument-specific pin configurations and achievable spot densities have already been created. If a suitable file is not available, an existing one should be downloaded and edited. An example file for the user to record their specific print cycle times is also available for editing. The 'index.sa' file should then be updated, to record the locations and names of the configuration files. The configuration files will then only need to be re-edited if the printing environment is altered.

### First run

**Probe number: **enter the number of microarray probes (or wells) to be printed.

**Plate format: **select an appropriate plate format.

**Tools available: **select an appropriate pin configuration.

**Source visits: **the source visit list is generated for editing.

### Second run

**Required spot density: **SimArray counts the number of spots to be printed.

**Pin type: **select the spotting pin to be used.

**Evaluate pin selection: **SimArray evaluates whether the selected pin is compatible with the required spot density.

**Compute spots_x and spots_y: **SimArray computes and displays the sub-grid dimensions that fall between the target spot number and a user-specified upper limit, for the user to select.

**Compute print time: **select an appropriate print set-up, SimArray then calculates the estimated print time.

**Summary report: **SimArray generates a report containing a summary of the user's responses, the randomised spot layout, an estimated print time, and the maximum meta-grid area.

### After the second run

The randomised source visit map can be directly uploaded to instruments that accept either comma-, tab-, or space-separated values source files, or manually entered. Microarrays can then be manufactured, with spots no longer positioned according to print order. Standard robotic spotter data tracking software can be used to record which probe is present at each spot location.

## Results and Discussion

**Figure 1:**
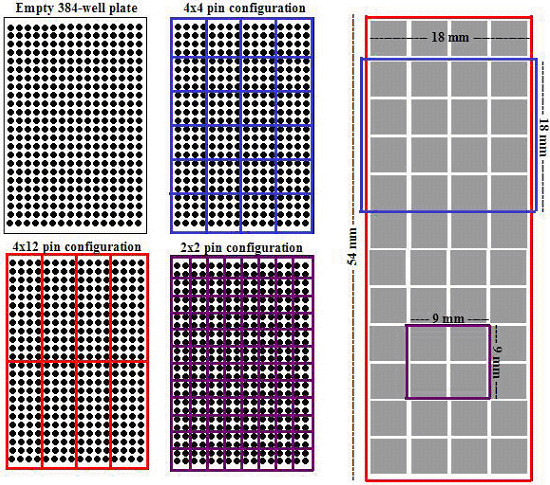
**Pin configuration directly affects array area and source visit number. **Each pin in the print tool will print a single patch or sub-grid of spots. The sum total of sub-grids printed by one print tool is called a meta-grid. Pin configuration directly affects maximum meta-grid area because the spotting pins have a fixed plate-specific pitch to enable adjacent pins in the tool to enter adjacent wells of the microtitre plates. 96-well print tools have a pitch of 9 mm, 384-well print tools have a pitch of 4.5 mm, and 1536-well print tools have a 2.25 mm pitch. SimArray estimates maximum meta-grid area by simply multiplying the number of pins in each axis by the pin pitch. SimArray will thus overestimate the meta-grid area, when a reduced spot pitch is employed because spot pitch is not taken into consideration. Pin configuration also defines how many spots need to be printed per sub-grid, as more pins means that fewer pin loadings or source visits need to be performed. For example, a 4 × 12 print tool will print all probes in a 384-well plate with just 8 source visits, meaning that each pin will print just 8 spots per sub-grid. Whereas, a 4 × 4 tool would require 24 source visits, and a 2 × 2 tool requires 96 source visits.

**Figure 2:**
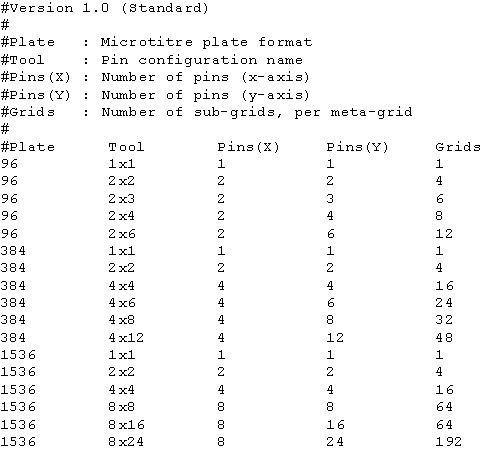
**Example 'tool' user-configurable file. **Print tools are composed of spotting pins that are arranged in a pre-defined plate-format-specific configuration (Fig. 1.). Each user records the pin configurations that are available to them by adding their robotic spotter's list of options to the tool file. Pin configuration directly affects source visit number and maximum meta-grid area (Fig. 1). Tool files for the Qarray2 (Genetix), MicroGrid, and OmniGrid (Genomic Solutions) robotic spotters are available from the SimArray web site [31].

**Figure 3:**
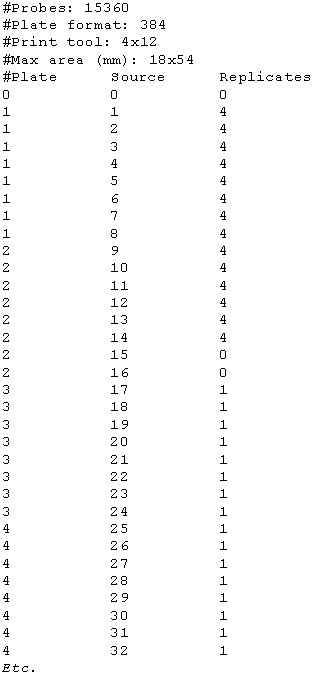
**Example SimArray source visit list. **The source list is generated after the first run of SimArray. Headers are marked with a hash at the beginning of the line and provide a summary of the specified design decisions. The replicate column can be edited to record how many times each source visit is to be printed. Source visits with zero replicates are ignored and omitted from the final array design. Other columns and the header should not be modified. An example file is available from the SimArray web site [31].

Pin configuration affects the number of source visits that must be performed and the maximum meta-grid area (Fig. 1). For these reasons, the user is required to enter the probe number (an integer) and to select a microtitre plate format (*e.g*., 96, 384 or 1536), before selecting a compatible pin configuration (Fig. 2). SimArray then prints a source visit list for the user to edit (Fig. 3). If the number of probes to be printed is not compatible with the selected pin configuration, SimArray will round up the source visit number to the nearest whole number, as robotic spotters can only print with a full complement of spotting pins. In such instances, the last source visit to be printed would include some empty wells. Users are, however, able to specify any number of replicates, for any number of source visits. Additionally, source visits can be omitted by setting the replicate number to zero. These features enable array designs with odd numbers of probes, variable numbers of replicates, and non-sequential source visits to be processed.

Maximum meta-grid area is calculated by simply multiplying the number of pins in each axis by the pin tool's pre-defined pin pitch (Fig. 1). Consequently, SimArray does not take spot pitch into consideration and can over-estimate meta-grid areas, especially for low-density arrays that are printed with reduced spot pitches. Since high-density arrays limit the scope for reducing spot pitch, we believe this is a reasonable approach because SimArray will be of most use when designing higher-density arrays. Additionally, most operators print microarrays with the spot pitch set to the near-maximum distance permissible to reduce the probability that neighbouring spots printed by the same pin will be merged together. Prediction of the maximum meta-grid area will at least allow users to decide whether it is possible for them to hybridise the array, *e.g*., when the hybridisation area is constrained by automated hybridisation stations.

**Figure 4:**
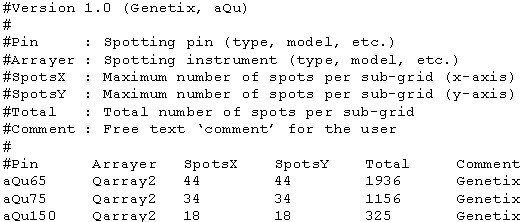
**Example 'pins' user-configurable file. **Achievable spot densities vary according to the production environment, *i.e*., spotting pin, spotting buffer, substrate, temperature, relative humidity, *etc*. The available pins and their achievable spot densities are recorded by editing the pins file. These data will directly affect selection of the sub-grid dimensions, *i.e*., the number of spots that the user would like to print in the x- and y-axis. Pin files for Telechem, Genetix, Matrix, and Genomic Solutions spotting pins are available from the SimArray web site [31].

The number of spots to be printed per sub-grid is calculated by counting the number of spots that are specified in the source visit list. This total is displayed and users are asked to select a suitable spotting pin (Fig. 4). The selected pin is evaluated and the script exits if the pin's achievable spot number per sub-grid is incompatible with the required target spot number per sub-grid. Exiting the script at this stage, if a problem is found, removes the risk of downstream errors and provides an opportunity for the array design to be modified, or for a different spotting pin to be selected.

Sub-grid dimensions, *i.e*., the number of spots in the x and y-axis, which are compatible with the target spot number per sub-grid are then calculated, and users select an option from a list of compatible choices. To limit the length of this list, SimArray will only display sub-grid dimensions that are equal to or greater than the target spot number per sub-grid and less than a user specified limit. The upper limit is the target spot number per sub-grid, plus the user-specified 'spot number margin'. SimArray prevents users from selecting grid dimensions that are incompatible with the spotting pins' maximum achievable spot density. If, however, the selected sub-grid dimensions permit more than the required number of spots to be printed, additional spot locations are flagged as blanks by assigning them a source visit number of zero, *i.e*., not printed. SimArray will fail at this stage if there are no viable sub-grid dimension between the minimum and maximum target. We therefore recommend using a 'spot number margin' of at least ten.

**Figure 5:**
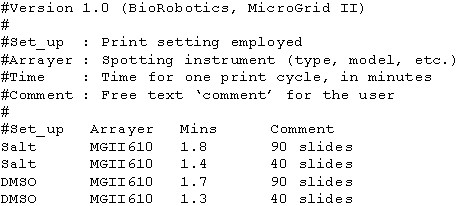
**Example 'time' user-configurable file. **Print time is dependent on a range of instrument and user-defined parameters. To estimate the print time, users record the time it takes to perform a single print cycle for a given print set-up by editing the time file. Single print cycle duration is best measured by recording how long it takes to perform a full print-run (under defined conditions) and then dividing by the number of the print cycles. Estimated print time is limited by the user-specified print cycle times. An additional example file is available from the SimArray web site [31].

Print time is dependent on the number of source visits (Fig. 1), the number of slides to be printed, a range of (perhaps) user-defined options, *e.g*., pre-blotting, contact speed, *etc*., and the hardware itself, *e.g*., microtitre plate handling, x-y-z-axis motor speeds, pin (or tool) travel distances, *etc*. Additionally, wash conditions vary according to the production environment, *i.e*., spotting pin, spotting buffer, *etc*. Print time is therefore calculated after the user has selected the intended print setting from a list of available options (Fig. 5). The list of options includes the user defined 'single print cycle duration', *i.e*., the time it takes to perform a single print cycle from source visit to wash/dry cycle. This has to be determined empirically because it is production environment and instrument dependent. Print time is calculated by simply multiplying the number of print cycles that need to be performed, by the time taken to perform each print cycle. Print time accuracy is therefore dependent on the user-specified single print cycle estimates.

**Figure 6:**
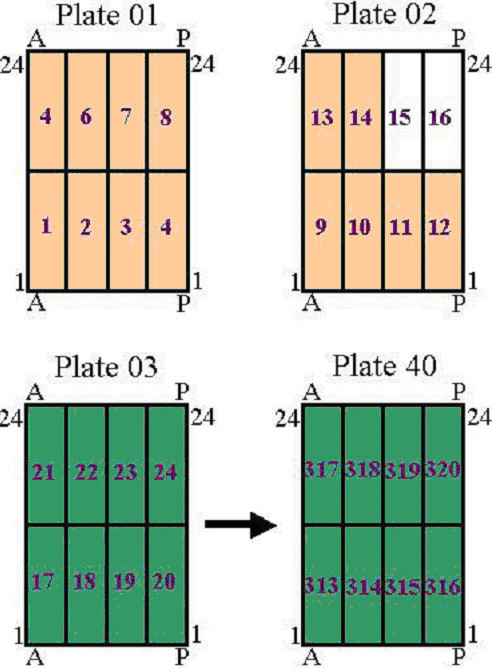
**Example library. **These probes are to be printed by a 48-pin print-head, with the pins arranged in a 4 × 12 configuration. The library consists of 14 independent exogenous controls (orange) and 14,592 transcript-specific probes (green). Since the exogenous controls must be printed by all pins, these must be arranged in 4 × 12 blocks of 48 wells. Conversely, the 14,592 target probes only need to be randomly distributed, to facilitate printing once per meta-grid and to ameliorate systematic biases [18]. Source visit number and hence print order are indicated in the figure.

Finally, a report containing the randomised source visit map along with a summary of the user's responses, an estimated print time, and the estimated maximum meta-grid area is generated (Fig. 6). The user can specify a comma-, tab, or space-separated source visit map with the command line keys -C, -T, or -S (default), respectively. The source visit map can then either be directly uploaded to instruments that accept source files in these formats, or manually entered. Microarrays can then be manufactured, with the spots and replicates positioned randomly, rather than according to their print order. Standard instrument data tracking software can be used to document what probe is present in each spot. New array designs are appended to the existing report to provide a full record of all array designs. Users are therefore able to perform multiple 'simulated print runs', with different configurations to compare the results, *i.e*., the estimated print times and maximum meta-grid areas. Each array design includes a date and time stamp.

The user-configurable files described above and in Figures 2, 3 and 4, maximise the utility of this tool because they enable a range of production environments to be explicitly modelled. All SimArray configuration files contain a header, which includes a key to the file's contents to help with this task. If required, additional comments can also be added because all lines marked with a hash at the start are automatically ignored when SimArray reads these files. However, column meaning and order is fixed and must be preserved. We aim to develop an on-line library of configuration files at the SimArray web site [31].

### A fully worked simple example

For this worked example, we compare the performance of a MicroGrid II (Genomic Solutions) and a Qarray2 (Genetix) instrument, printing a 15 K probe library. The configuration files were edited to reflect the specific set of printing conditions for each robotic spotter. The example library consists of two probe types: transcript-specific probes and exogenous controls, along with some empty wells (Figure 6). The design requirement is to print single copies of the transcript-specific probes, and for every pin to print quadruplet spots for the exogenous control probes, randomising the distribution of elements on the array, whilst omitting the empty wells. The probes were arranged in the spotting plates, according to these design criterion (Figure 6).

**Figure 7:**
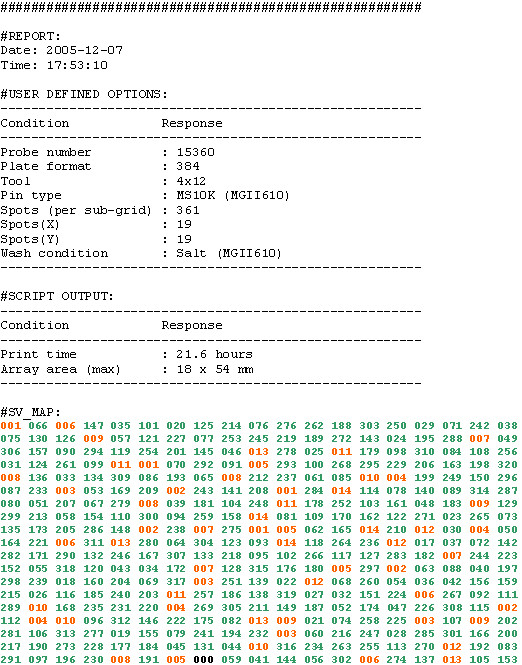
**Example SimArray summary report. **The report is generated after the second run of SimArray and includes a summary of the user-specified design decisions, estimated print run time, estimated maximum meta-grid area, and the randomised source visit map. The source visit map can be either comma,- tab- or space-delimited (default). In this example, and in Figure 8, the design requirement was to print 4 × copies of the exogenous spike control source visits (orange), 1 copy of each transcript-specific probe source visit (green), and omit the empty wells. This report was generated whilst simulating printing by a MicroGrid II 610 robotic spotter (Genomic Solutions). This figure has been colour-coded to aid comparison with Figures 6 and 8.

**Figure 8:**
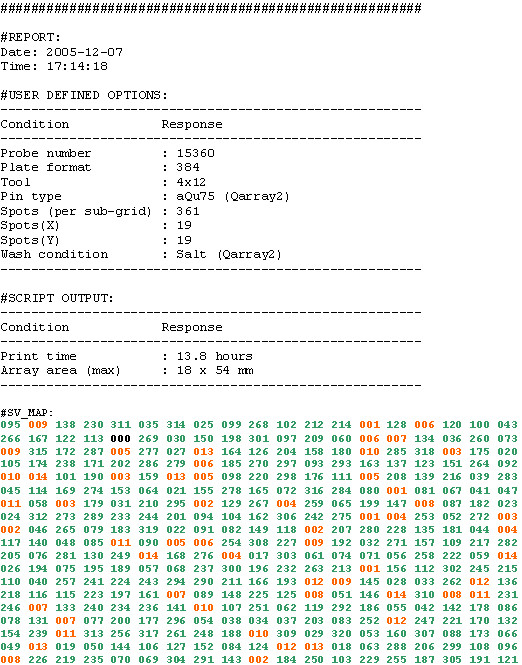
**Alternative SimArray array report. **The parameters described in Figure 7 were used to simulate printing the library described in Figure 6 with a Qarray2 (Genetix). In this case, the print time has reduced from almost 22 hours to 14 hours, showing that either the Qarray2 is the faster instrument or the MicroGrid II print conditions are not optimal. This example demonstrates that SimArray rapidly facilitates refinement of microarray designs, whilst generating randomised source visit maps that can be used to program robotic spotters. This figure has been colour-coded to aid comparison with Figures 6 and 7.

SimArray was used to generate a randomised spot layout for each instrument, to assess which would be better suited to printing this library. The SimArray simulated print-runs indicated that the MicroGrid II spotter would take 56% longer to print microarrays according to the specified criterion (Figs 7 and 8). The estimated print times agreed with how long it would take to print the arrays, provided no manual intervention, *e.g*., refilling of wash solutions, *etc*., was required. The user can now either enter the randomised spot layout and print this microarray design with the Qarray2, or re-evaluate whether the print settings for the MicroGrid II were optimal.

SimArray also permits further simulations, allowing an evaluation of alternative pin configurations, replicate numbers, slide numbers and instrument configurations, subject to the availability of appropriately annotated configuration files. This further ensures that microarrays of an ideal design can be generated, whilst permitting each to be evaluated for its compatibility to the local production environment. Printing with different pin configurations, however, requires the spotting library itself to be redesigned, as spotting pins enter adjacent wells of the microtitre plate and the probes must be arranged accordingly (Figs 1 and 6). This suggests that spot layouts should be defined before the spotting probes are transferred to microtitre plates for printing.

## Conclusion

We have developed a user-friendly microarray design tool, SimArray, which generates a randomised spot layout, computes a maximum meta-grid area, and an estimated print time, in response to user-specified design decisions. SimArray is of general utility for all users of robotic spotters and can be configured to suit individual production environments.

## Availability and requirements

**Project name: **SimArray

**Project home page: **

**Operating system: **Windows and UNIX

**Programming language: **Perl version 5.6.1 (and more recent versions)

**Other requirements: **tool (Fig. 2), pins (Fig. 4), and time (Fig. 5) configuration files

**Licence: **free

**Any restrictions to use by non-academics: **none

## List of abbreviations used

cDNA Complementary DNA

gDNA Genomic DNA

Meta-grid The sub-grids (and spots) that are printed by one print tool

Sub-grid The patch of spots that is printed by a single pin

## Authors' contributions

RRR suggested that array design could be automated. RPA wrote the source code, with technical guidance from RRR. RPA, BF, LAM, and SSM tested the code and validated performance. The manuscript was prepared by RPA. SR is the group leader, providing funding, critical assessment and general guidance. All authors read and approved the final manuscript.

## Supplementary Material

Additional File 1 This dataset includes heat maps for six different microarrays that were printed and hybridised by the UK *Drosophila *microarray facility. The heat maps demonstrate that spot location has a direct impact on the measured differential gene expression ratios and hence supports the argument for randomisation of the spot layout.
Click here for file
