# Supplementary material for: SimArray: a user-friendly and user-configurable microarray design tool
Source: BMC Bioinformatics. 2006 Mar 1;7:102. doi: 10.1186/1471-2105-7-102 (PMC1456992; doi:10.1186/1471-2105-7-102)
Supplement: Additional File 1 — This dataset includes heat maps for six different microarrays that were printed and hybridised by the UK Drosophila microarray facility. The heat maps demonstrate that spot location has a direct impact on the measured differential gene expression ratios and hence supports the argument for randomisation of the spot layout. [file 1471-2105-7-102-S1.doc]

## Spatial bias heat maps

Spatial variation is observed even in the best laboratories [1-5]. Spatial bias can be plotted in form of heat maps. The following heat maps were selected at random, and show six dual-channel microarrays. The microarrays were printed and hybridised according to the protocols given by the UK *Drosophila* microarray facility web site [6]. The x/y-coordinates represent location on the microarray, whilst the colouring and contours indicate the difference between the Cy5 and Cy3 channels. These six examples show that spot location has a direct impact on the measured differential gene expression ratios and hence supports the need for randomisation of the spot layout to facilitate correction of spatial biases by normalisation [7-11].

# First example

Two patches of high gene expression ratios towards the centre of the array and decreased ratios towards the edges, especially the right edge. The top and bottom are roughly equivalent to the central region.


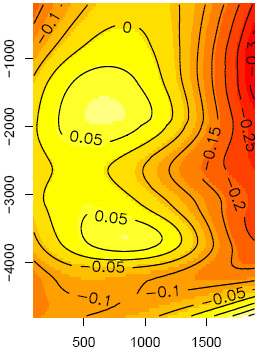


# Second example

Gradual increase in the measured gene expression ratios from bottom-to-top. The rate of increase varies between the left and right, leading to a second gradient running across the centre of the array from right-to-left.


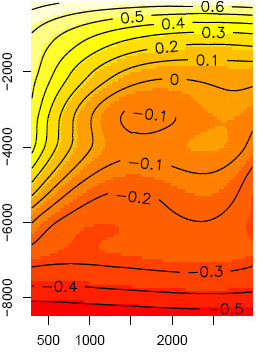


# Third example

Low gene expression ratios at the centre, top and bottom of the array. Higher ratios towards the left and right edges.


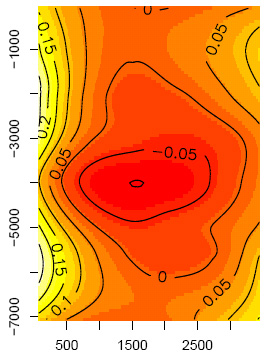


# Fourth example

Low ratios at the left and right edges that appear to increase towards the centre and then again at the top of the microarray.


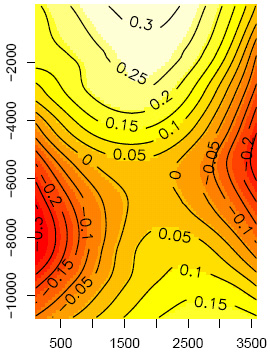


# Fifth example

High ratios at the bottom and top-left corner of the array, low ratios for one patch on the left edge and the entire top-right corner.


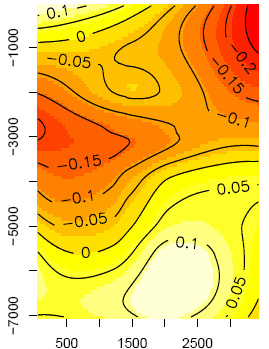


# Sixth example

Reasonably even ratios across most of the microarray, but with slightly higher ratios in the top-left corner.


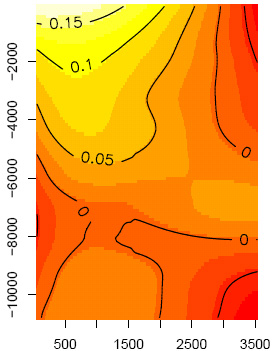


## References
